# Sweet Taste Receptors’ Genetic Variability in Advanced Potential Targets of Obesity

**DOI:** 10.3390/nu17101712

**Published:** 2025-05-18

**Authors:** Sonia Wagner-Reguero, Lara P. Fernández, Gonzalo Colmenarejo, Silvia Cruz-Gil, Isabel Espinosa, Susana Molina, María Carmen Crespo, Elena Aguilar-Aguilar, Helena Marcos-Pasero, Rocío de la Iglesia, Viviana Loria-Kohen, Ricardo Ramos Ruiz, Moisés Laparra-Llopis, Ana Ramírez de Molina, Marta Gómez de Cedrón

**Affiliations:** 1 Molecular Oncology Group, IMDEA Food Institute, CEI UAM+CSIC, 28049 Madrid, Spain; 2 Biostatistics and Bioinformatics Unit, IMDEA Food CEI UAM+CSIC, 28049 Madrid, Spain; 3 Nutrition and Clinical Trials Unit, GENYAL Platform IMDEA-Food Institute, CEI UAM+CSIC, 28049 Madrid, Spain; 4 Department of Pharmacy and Nutrition, Faculty of Biomedical and Health Sciences, Universidad Europea de Madrid, 28670 Madrid, Spain; 5 Food and Nutrition in Health Promotion (CEU-NutriFOOD), Departamento de Ciencias Farmacéuticas y de la Salud, Facultad de Farmacia, Universidad San Pablo-CEU, CEU Universities, Urbanización Montepríncipe, 28660 Boadilla del Monte, Spain; 6 Department of Nutrition and Food Science, Faculty of Pharmacy, Complutense University of Madrid, 28040 Madrid, Spain; 7 Molecular Immunonutrition Group, IMDEA Food Institute, CEI UAM+CSIC, 28049 Madrid, Spain

**Keywords:** Precision Nutrition, obesity, sweet taste signaling, nutrigenetics, gene-diet interactions

## Abstract

*Background*: Obesity, mainly visceral obesity, causes a low-grade of chronic inflammation (meta-inflammation), associated with comorbidities such as type 2 diabetes, cardiovascular diseases, and certain cancers. Precision Nutrition aims to understand the bidirectional crosstalk between the genome and diet to improve human health. Additionally, by leveraging individual data, Precision Nutrition seeks to predict how people will respond to specific foods or dietary patterns, with the ultimate goal of providing personalized nutritional recommendations tailored to their unique needs and lifestyle factors, including poor dietary habits (e.g., high intake of sugar or saturated fatty acids, alcohol consumption, etc.) and sedentary habits, exacerbate obesity in genetically predisposed individuals. Genetic, metabolic, and environmental factors can play a crucial role during obesity. *Objective*: To investigate the effects of genetic variability in sweet taste receptors and their downstream signaling pathways in the gut–brain axis on anthropometry, biochemistry, and lifestyle variables. *Methods*: A sample of 676 volunteers (mean age of 42.22 ± 12 years, ranging from 18 to 73 years) from the database of the GENYAL platform for nutritional trials at the IMDEA Food Institute were included in this study. We present a first-in-class genetic chip, Glucosensing, designed to interrogate 25 single-nucleotide polymorphisms (SNPs) located in genes encoding sweet taste receptors and components of downstream signaling pathways. These include elements of the gut–brain axis and its associated metabolic networks, enabling a comprehensive analysis of individual variability in sweet taste perception and metabolic responses. *Results*: Several significant associations were found after correction for multiple comparisons, representing potential targets for personalized interventions.

## 1. Introduction

Obesity is a global epidemic affecting over 2.5 billion adults which significantly increases the risk of comorbidities such as type 2 diabetes, cardiovascular diseases, and certain types of cancers. Precision Nutrition aims to understand the bidirectional crosstalk between the genome and diet to improve human health. Additionally, by leveraging individual data, Precision Nutrition seeks to predict how people will respond to specific foods or dietary patterns, with the ultimate goal of providing personalized nutritional recommendations tailored to their unique needs [1,2,3]. Additional factors associated with the lifestyle characteristics and nutritional and metabolic status of individuals (e.g., obesity, metabolic syndrome, dyslipidemia, etc.) are key factors to be considered in the development of personalized interventions in chronic diseases [4]. The genetic contribution to obesity has been extensively investigated in genome-wide association studies (GWAS) which have successfully identified susceptibility loci linked to obesity [5,6,7]. Furthermore, other habits, such as fried food consumption [8], high intake of saturated fatty acids [9], sleep disturbances [10], and sedentary lifestyles [11,12,13], also interact with genetic variants in obesity association studies [14,15,16]. A recent study described a positive association between sugar-sweetened beverages consumption and body mass index (BMI) in individuals genetically predisposed to obesity [17].

While the primary factor in the development of obesity is the energy balance—specifically, a chronic energy surplus where energy intake consistently exceeds energy expenditure—it also should consider the role of genetic variations in this context. A systematic review of observational studies has highlighted genetic determinants that influence food preferences, which are closely associated with obesity. This genetic predisposition may pose additional challenges for certain individuals in maintaining a balanced energy intake [18].

When we consider sweet taste receptors, we typically focus on the oral cavity; however, it is worth noting that sweet taste receptors have been identified in other organs, including the gastrointestinal (GI) tract, pancreas, adipose tissue, skeletal muscle, bladder, and brain [19]. Several studies have explored the role of sweet taste receptors (TASR) in the regulation of physiological functions at the GI tract, including the regulation of GI motility and the secretion of enterohormones (such as leptin, ghrelin, insulin, GLP-1, and endocannabinoids) closely involved in energy balance, glucose levels, and food intake [20,21].

Sweet taste sensitivity is influenced by genetic variations in taste receptors, particularly in the *TAS1R2* and *TAS1R3* genes. The gut–brain axis is a bidirectional communication system between the GI tract and the central nervous system (CNS). It involves various signaling molecules, including hormones, neurotransmitters, and microbial metabolites. It is not well known to what extend genetic variations in sweet taste receptors may affect sweet taste sensitivity and the release of incretins and enterohormones involved in appetite regulation and glucose homeostasis. Nevertheless, recent studies have shown that individuals with heightened sweet taste sensitivity exhibit altered GLP-1 secretion, which, in turn, affects insulin sensitivity and glucose metabolism [22,23,24].

Variations in genes encoding sweet taste receptors, such as *TAS1R2* and *TAS1R3*, influence individuals’ perceptions of sweetness and fat, potentially affecting dietary choices and contributing to increased fat storage and obesity. One study investigated single-nucleotide polymorphisms (SNPs) in *TAS1R2* and *TAS1R3*, revealing associations between specific variants and body mass index (BMI) [25]. The study concluded that individuals carrying certain genetic variants exhibited reduced sensitivity to sweet and fatty tastes, which may lead to increased consumption of sugary and high-fat foods, thereby promoting the development of obesity. In a separate study, individuals with specific polymorphisms in these genes demonstrated varying levels of sweet taste sensitivity and carbohydrate intake, further supporting a genetic predisposition to obesity [26].

Furthermore, the metabolic pathways influenced by sweet taste sensitivity are closely linked to the regulation of fat storage. Activation of sweet taste receptors in the gut modulates the expression of genes involved in lipogenesis and adipogenesis. For example, the sterol regulatory element-binding protein 1c (SREBP-1c) pathway is regulated by dietary sugars through sweet taste receptor signaling. Genetic variations that enhance sweet taste sensitivity have been associated with increased fat storage, thereby contributing to the development of obesity [27,28]. There is considerable interindividual variation in sweet taste perception and dietary preferences. Recent studies have demonstrated that variations in *T1R* genes not only influence food choices and intake, but are also associated with other behavioral traits, such as the proclivity for alcohol consumption [29]. All of these data illustrate the complex genetics of sweet taste preferences and its impact on human nutrition and health.

The objective of this study is to investigate how genetic variants in sweet taste receptors and downstream signaling pathways are associated with various phenotypic outcomes, including anthropometry, biochemical markers, dietary patterns, and lifestyle choices. Understanding these associations is crucial for elucidating the complex interplay between genetics and metabolic health. A glucosensing genetic chip was designed with 25 SNPs along the genes of sweet taste receptors and related pathways, such as glucose uptake and gut–brain axis signaling (e.g., incretin secretion and enterohormone signaling). A total of 676 volunteers (mean age of 42.22 ± 12 years, ranging from 18 to 73 years) from the database of the GENYAL platform for nutritional trials at IMDEA Food Institute were included in the study. Several significant associations were found after correction for multiple comparisons, representing potential targets for personalized interventions.

## 2. Materials and Methods

### 2.1. Study Design

A sample of 676 volunteers was selected from the database of the GENYAL Platform for Clinical Trials in Nutrition and Health at the IMDEA Food Institute (Madrid, Spain). This study is part of a series of investigations conducted within the GENYAL Platform. Ethics committee approval numbers are provided in the Appendix A. All procedures were conducted in accordance with the principles outlined in the Declaration of Helsinki (1964) and Good Clinical Practice (GCP) guidelines.

Inclusion criteria included an adequate understanding of the research protocol and prior provision of informed consent for the use of biological samples in future studies.

The main objective of this study was to investigate the associations between genetic variants in sweet taste receptors and downstream signaling pathways at the gastrointestinal level and anthropometric measurements, biochemical parameters, dietary patterns, and lifestyle factors.

Anthropometric measurements were obtained using standardized and validated techniques. Body weight was measured using the BF511 body composition monitor (Omron Healthcare UK Ltd., Kyoto, Japan). Height was assessed using a stadiometer (Leicester–Biological Medical Technology SL, Barcelona, Spain), and waist circumference was measured using a non-elastic Seca 201 tape (Quirumed, Valencia, Spain).

Dietary patterns were assessed using a validated 72 h dietary food record and a food frequency questionnaire (FFQ). Energy intake, Healthy Eating Index (HEI), and macro- and micronutrient intake were calculated using DIAL software (version 2.16, Alce Ingeniería, Madrid, Spain) [30].

Lifestyle data were collected, including physical activity (categorized as inactive: 0 sessions/week; active: ≥1 session/week), smoking habits (cigarettes/day), and alcohol consumption.

Biochemical analyses included the quantification of glucose (mg/dL), total cholesterol (mg/dL), HDL (mg/dL), LDL (mg/dL), triglycerides (mg/dL), insulin (ng/dL), leptin (ng/dL), GOT (U/L), GPT (U/L), IL-6 (ng/L), IL-8 (ng/L), IL-1β (ng/L), TNF-α (ng/L), and APOA1 (mg/dL).

Quality of life was assessed using the validated SF-36 questionnaire, which evaluates multiple health-related domains.

To minimize potential bias in self-reported dietary and lifestyle data, participants received detailed instructions on how to accurately record their intake and behaviors. These instructions included guidance on portion sizes, food preparation methods, and the importance of accurate and honest reporting. Participants were encouraged to use standardized measuring tools (e.g., measuring cups, food scales) to improve precision. All questionnaires and anthropometric measurements were administered by the same trained personnel to ensure consistency in data collection.

**Figure 1:**
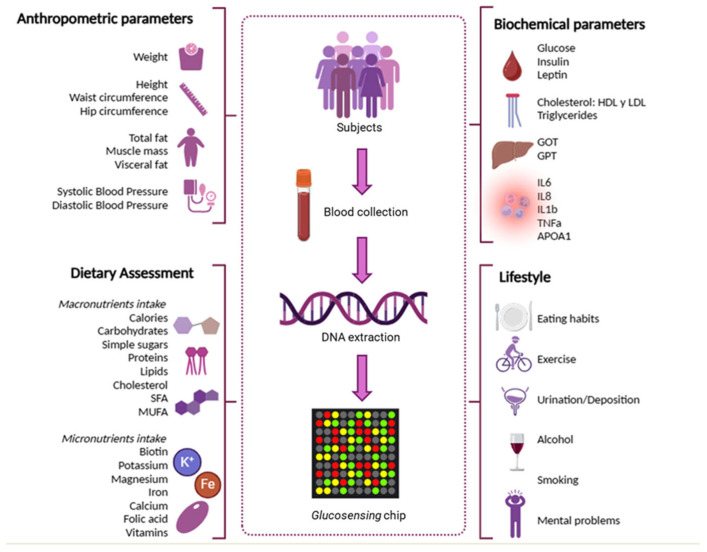
Study design indicating the main variables considered -anthropometric measurements, dietary intake (macro- and micronutrients), lifestyle factors (alcohol consumption, smoking, physical activity), and biochemical biomarkers.

Figure 1 illustrates the study design, including the main variables assessed: anthropometric measurements, dietary intake (macro- and micronutrients), lifestyle factors (alcohol consumption, smoking, physical activity), and biochemical biomarkers.

### 2.2. Descriptive Data of the Sample

A total of 676 volunteers from the GENYAL Platform for Clinical Trials in Nutrition and Health were included in the study. The participants had a mean age of 42.22 years (SD ± 12), with an age range of 18–73 years.

Anthropometric data were collected by trained nutritionists at the IMDEA Food Institute, while dietary and lifestyle data were obtained using validated questionnaires, including the SF-36, MEDAS, and a 72 h dietary intake record. Biochemical data were derived from analyses conducted as part of each participant’s respective study within the platform.

#### 2.2.1. Anthropometric Measurements and Vital Constants

Anthropometric characteristics and vital signs of the sample, stratified into three groups based on body mass index (BMI: <25, 25–30, and >30), are summarized in Table 1A. The minimum BMI recorded was 18.2, corresponding to the lower limit of the normal weight range, while the maximum was 43.4, classified as grade III obesity. The mean BMI of the total sample was above 25, placing the population in the overweight category.

Visceral fat, assessed via bioelectrical impedance analysis (BIA), had a mean value of 8.57, which is below the threshold considered detrimental to health. Body circumferences, which vary according to sex and body morphology, were generally within normal limits. Regarding vital signs, mean blood pressure values were within the normal physiological range.

#### 2.2.2. Dietary Assessment

The dietary assessment included the evaluation of total macronutrient intake, as presented in Table 1B, which reflects the daily nutrient consumption reported by participants in their dietary records, alongside the corresponding recommended daily intakes. Macronutrients are expressed as a percentage of total energy intake (expressed in calories). The mean intake of carbohydrates and sugars in the total sample was significantly higher than the recommended values, as was the intake of other macronutrients. These findings suggest that the general dietary pattern of the study population was unbalanced, with an excessive intake of certain components.

Micronutrient intake, also shown in Table 1B, was compared with the recommended dietary allowances (RDA). In general, the population met the RDA for most micronutrients evaluated, except for biotin, vitamin B3, and vitamin D, for which intake levels were below the recommended thresholds.

#### 2.2.3. Biochemical Parameters

Biochemical parameters are summarized in Table 1C, including both the mean values obtained from laboratory analyses and the corresponding reference ranges.

Overall, the study population exhibited biochemical health markers within normal limits, with a few parameters approaching the upper threshold of the healthy range. However, these deviations were not clinically significant.

#### 2.2.4. Lifestyle

Most participants reported consuming home-prepared meals during the week, with an average of 1–2 meals eaten outside the home on weekends. Water intake was significantly below recommended levels, with participants consuming less than 2 L per day on average. The mean glycemic index (GI), calculated based on carbohydrate and sugar intake, was above 70 for the total sample, indicating a high-GI dietary pattern.

Regarding physical activity, 36.92% of participants reported moderate activity, 29.46% reported low activity, and 33.62% reported high activity levels. Table 1D summarizes the frequency of physical activity, along with data on alcohol consumption, bowel habits, and urinary frequency.

Overall, most participants engaged in physical activity two or more times per week and reported adequate gastrointestinal function. Alcohol consumption was reported daily by 67% of participants, while the majority were non-smokers, as shown in Table 1E.

In terms of mental health, 25% of participants reported experiencing stress, 15% reported anxiety, and less than 1% reported symptoms of depression.

### 2.3. Systematic Search for Gene Selection of the Glucosensing Chip

To select the SNPs to be included in the glucosensing genetic chip, a systematic search was conducted, including the open-access PubMed database (www.ncbi.nlm.nih.gov/PubMed, accessed on October–December 2021), the GeneCards database (www.genecards.org, accessed on October–December 2021), and the single-nucleotide polymorphism (SNP) database (dbSNP-Short Genetic Variation) from the National Center for Biotechnology Information: NCBI (www.ncbi.nlm.nih.gov/snp, accessed on October–December). The latter database was used to confirm the location in the sequence and the influence of each polymorphism on amino acid changes.

A total of 25 SNPs were selected based on their involvement in metabolic pathways related to sweet taste receptors and downstream signaling pathways at the gut–brain axis. For refining the search for SNPs involved in the pathways of interest, the following criteria were considered, with the last two being exclusionary: (1) SNP location—priority was given to intragenic SNPs, specifically those located in coding regions. SNPs in the gene-promoter region (up to 10 kb before the transcription initiation site) and the final region (up to 2 kb after the transcription termination site) were also included, as well as some exonic SNPs of interest. (2) Not being in Linkage Disequilibrium (LD): The HapMap Project (http://hapmap.ncbi.nlm.nih.gov/, accessed on October–December 2021) was used to address the linkage disequilibrium (LD) maps, with the objective of identifying regions inherited together due to the low recombination frequency. The HapMap data for the Caucasian population were used, selecting only SNPs which were not in LD. The Haploview software (http://www.broad.mit.edu/mpg/haploview, accessed on October–December 2021) was used to analyze HapMap data. (3) Allelic frequency: The allele frequency in the Caucasian European population had to be greater than 0.1, meaning at least 10 out of 100 individuals would have the SNP.

### 2.4. Genotyping of the Sample

For genotyping, DNA samples were loaded onto TaqMan OpenArray real-time PCR plates (Life Technologies Inc., Carlsbad, CA, USA), pre-configured with specific probes for each SNP allele labeled with different fluorophores to determine the genotype. This process was conducted using the OpenArray AccuFill system (Life Technologies Inc., Carlsbad, CA, USA). Once loaded, PCR was performed, and the chips were read on the QuantStudio 12K Flex real-time PCR instrument (Life Technologies Inc., Carlsbad, CA, USA). Results were analyzed using TaqMan Genotyper software v.1.0.1 (Life Technologies Inc., Carlsbad, CA, USA), which automatically assigned genotypes to each sample based on the fluorophore signal detected.

### 2.5. Statistical Analysis

Statistical analysis was performed using R 4.1.2. Descriptive analyses of numerical variables included means and standard deviations, while medians and interquartile ranges were used for non-normally distributed variables. Categorical variables were analyzed using absolute and relative frequencies of their respective categories, and the number of available data points was determined for each variable.

For SNP analysis, Hardy–Weinberg equilibrium was tested, and LD between all SNPs was calculated. None of the SNPs significantly deviated from equilibrium, as expected from a genetically well-mixed sample. The association of each of these SNPs with different anthropometric, biochemical, and lifestyle variables was modeled using linear models adjusted by age and sex; this was required since the sample was not balanced with respect to these variables. Three different models were derived for each SNP: additive, dominant, and codominant. The *p*-value for the SNP variable was derived in each case, and multiple-test corrected by applying Bonferroni method. In all the statistical tests, a two-tailed significance level of 0.05 was used for inference, and confidence intervals were derived for 95% confidence.

Since this was an exploratory analysis, we did not attempt to make power and sample size calculations and instead tried to collect the largest sample possible.

## 3. Results

Single-nucleotide polymorphisms in genes encoding sweet taste receptors and associated downstream signaling may also be candidates of low-penetrance variants with a role in susceptibility to obesity development, which is also linked to lifestyle characteristics.

### 3.1. Design of the Glucosensing Chip

**Figure 2:**
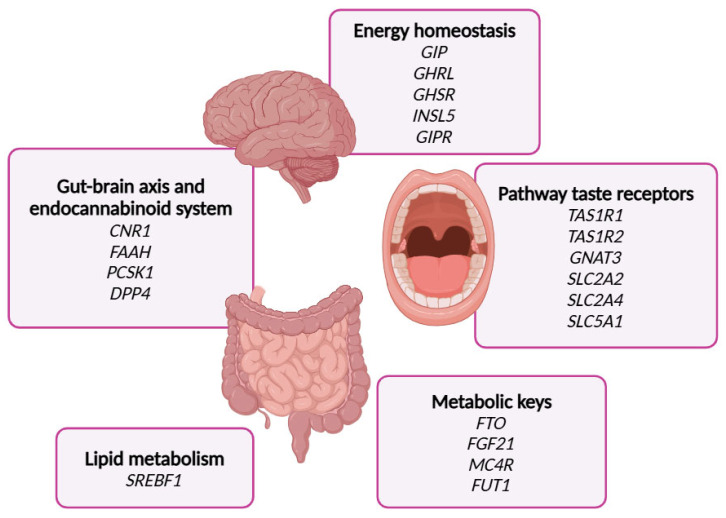
This Figure indicates the genes selected in the glucosensing chip including the metabolic processes where they are implicated.

First, an exhaustive bibliographic search was conducted to select genes of the sweet taste signaling at the gastrointestinal tract. A total of twenty genes were selected, including six genes from the taste receptors family (*TAS1R1*, *TAS1R2*, *GNAT3*, *SLC2A2*, *SLC2A4*, and *SLC5A1*); four genes linked to the gut–brain axis and the endocannabinoid system (*PCSK1, DPP4*, *CNR1*, and *FAAH*); six genes associated with hormones regulating energy homeostasis (*GHRL*, *GR INSL5*, *GIP*, and *GIPR*); three genes acting as metabolic regulators (*FGF21*, *FTO*, and *MC4R*); *SREBF1*, associated with lipid metabolism; and *FUT1*, whose mutations have been directly related to the consumption of sweet foods (Figure 2).

Based on the criteria described in the Materials and Methods section, a total of 25 single-nucleotide polymorphisms (SNPs) were selected for genotyping. To the best of our knowledge, this is the first study to design a custom genetic chip specifically aimed at exploring sweet taste perception and its associated downstream signaling pathways within the gut–brain axis.

Table 2 lists the selected SNPs, while Table 3 provides detailed information on their chromosomal allocation, genotype frequencies (%), minor allele frequencies (MAF), and Hardy–Weinberg equilibrium (HWE) status within the study population.

### 3.2. Genetic and Phenotypic Associations in the Sample

As indicated in the Study Design subsection, a total of 676 volunteers from the GENYAL Platform for Clinical Trials in Nutrition and Health were included in this study. The participants had a mean age of 42.22 years (SD ± 12), with an age range of 18–73 years.

Anthropometry: The anthropometric characteristics and vital signs included in the analysis were weight (kg), height (cm), BMI, total fat percentage, muscle mass percentage, visceral fat (measured by BIA), waist and hip circumferences (cm), systolic blood pressure (SBP, mmHg), and diastolic blood pressure (DBP, mmHg).

Diet: Dietary assessment involved estimating macronutrient and micronutrient intake based on the daily food records provided by participants. Although sugar consumption was a primary focus, additional parameters such as appetite sensations and eating habits were also considered to estimate the glycemic index (GI), which reflects the rise in blood glucose levels following the intake of specific carbohydrates.

Lifestyle: Lifestyle variables included the frequency of home-prepared meals, water intake, physical activity levels (low, moderate, and high), alcohol consumption, bowel and urinary habits, and mental health indicators such as anxiety, stress, and depression.

The analysis of the 25 SNPs included in the glucosensing chip, in relation to anthropometric, biochemical, and lifestyle parameters, revealed several statistically significant associations, which are summarized in Table 4.

A detailed description of the main association found is shown below.

The SNP rs1049353 in the *CNR1* gene was significantly associated with the glycemic index (GI). Thus, the minor allele (A) was associated with a lower GI under the additive model (Beta = −4.23, 95% CI: −6.8 to −1.67, *p*_adj_ = 0.033).

The SNP rs12617656 in *DPP4* showed several associations with total food intake and specific micronutrients obtained from the diet. Thus, the minor allele C was associated with a significant reduction in total food consumption, using the additive model (Beta = −132, 95% CI: −205 to −60, *p*_adj_ = 0.009); lower intake of several micronutrients -pantothenic acid (Beta = −0.388, 95% CI: −0.581 to −0.194, *p*_adj_ = 0.002); magnesium (Beta = −19.9, 95% CI: −30.7 to −9.04, *p*_adj_ = 0.009); potassium (Beta = −183, 95% CI: −283 to −83.6, *p*_adj_ = 0.009); and folic acid (Beta = −21.8, 95% CI: −34 to −9.72, *p*_adj_ = 0.011); reduced carbohydrate intake (Beta = −12.3, 95% CI: −19.1 to −5.42, *p*_adj_ = 0.012); and simple sugar intake (Beta = −6.76, 95% CI: −10.6 to −2.96, *p*_adj_ = 0.013).

The SNP rs2297508 in *SREBF1* positively associated with higher dietary intake of riboflavin (Beta = 0.129, 95% CI: 0.0597 to 0.198, *p*_adj_ = 0.007) and iron (Beta = 0.79, 95% CI: 0.313 to 1.27, *p*_adj_ = 0.031) under the additive model.

The SNP rs1800437 in *GIPR* showed an association with legume intake under the codominant model. The homozygous genotype (AA) was associated with an increase in legumes intake (Beta = 60.4, 95% CI: 29.3 to 91.5, *p*_adj_ = 0.012). Interestingly, allele A was positively associated with physical exercise (Beta = 0.383, 95% CI: 0.144 to 0.622, *p*_adj_ = 0.044).

The SNP rs324420 in *FAAH* was associated with TNFα levels, being the homozygous genotype (AA) for the minor allele associated with increased levels (Beta = 3.47, 95% CI: 1.59 to 5.35, *p*_adj_ = 0.039).

The SNP rs34160967 in *TAS1R1* was associated with an increased risk of being overweight and with the use of antidepressants. Individuals with at least one copy of the minor allele (A) showed a positive association with a higher risk of being overweight compared to the homozygous genotype for the reference allele (Beta = 0.522, 95% CI: 0.345 to 0.79, *p*_adj_ = 0.055). In addition, each minor allele was associated with higher use of antidepressants (Beta = 0.102, 95% CI: 0.00569 to 0.478, *p*_adj_ = 0.027).

The SNP rs3809770 in *GIP* was positively associated with increased alcohol consumption (Beta = 2, 95% CI: 0.763 to 3.24, *p*_adj_ = 0.041).

The SNP rs838133 in *FGF21* showed an association with hip circumference under the codominant model, where the heterozygous genotype was associated with decreased hip circumference (Beta = −2.48, 95% CI: −4.62 to −0.344).

Finally, the SNP rs9609429 in *SLC5A1* was associated with increased bowel motility under the additive model (Beta = 0.121, 95% CI: 0.0459 to 0.196, *p*_adj_ = 0.042).

## 4. Discussion

Herein, we present a first-in-class genetic chip specifically designed to explore the associations between single-nucleotide polymorphisms (SNPs) in sweet taste receptors and their downstream signaling pathways, including components of the gut–brain axis, in relation to anthropometric and lifestyle variables.

Sweet taste receptors are expressed not only in the oral cavity, but also in several extraoral tissues, including the gastrointestinal (GI) tract, pancreas, adipose tissue, skeletal muscle, urinary bladder, and brain, where they play important roles in nutrient sensing, metabolic regulation, and energy homeostasis [19]. These receptors regulate physiological functions at the GI level, including GI motility and the secretion of enterohormones (leptin, ghrelin, insulin, GLP-1, and endocannabinoids), being involved in energy balance, systemic glucose levels, and food intake [20,21,59]. One study compared insulin levels after oral vs. intravenous glucose administration, observing that insulin levels were higher when glucose was administered orally compared to intravenously, revealing an effect of the glucose sensing at the GI level on systemic insulin secretion. Conversely, the inhibition of TAS1R2-TAS1R3 with lactisole reduced the secretion levels of GLP-1 and GIP incretins [60]. Obese individuals have been shown to present reduced expression levels of the sweet taste receptor TAS1R3 [61], suggesting a decreased glucose sensing in these individuals. Similarly, individuals with T2DM experience reduced expression levels of the sweet taste receptors TAS1R2 and TAS1R3, as well as their regulator, α-gustducin [62]. Overall, the manipulation of sweet taste receptor responses at the GI tract is a promising field for developing therapeutic approaches against obesity and T2DM.

The sweet taste signaling pathway in the gastrointestinal (GI) tract exerts not only local effects, but also systemic effects through the gut–brain axis, playing a key role in the regulation of satiety and dietary preferences [63]. The endocannabinoid system, which includes the CNR1 and CNR2 receptors, is involved in the regulation of energy homeostasis, appetite, and body weight [64,65].

The SNP rs1049353 in the CNR1 gene has been associated with obesity [32], type 2 diabetes mellitus (T2DM) [66], and metabolic syndrome [67]. Meta-analyses have shown that individuals with GA/AA genotypes tend to have a lower BMI compared to those with the GG genotype [68]. This polymorphism is relatively common, with a 48.1% frequency of the minor allele (A) in the Spanish population [69]. Our findings regarding glycemic index (GI) values are consistent with the previous literature [70,71,72], reinforcing the association between the rs1049353 SNP and improvements in glucose metabolism, as well as reductions in fat mass, body weight, and BMI [73].

Dipeptidyl peptidase-4 (DPP4) is a multifunctional protein with peptidase activity on substrates like GLP-1 and GIP, being involved in hyperglycemia, insulin resistance, dyslipidemia, oxidative stress, and inflammation [74,75,76,77,78]. Polymorphisms in the *DPP4* gene have been linked to T2DM and myocardial infarction [33,79]. Minor alleles of SNPs rs12617336 and rs17574 are protective against hypoalphalipoproteinemia, insulin resistance, and hyperinsulinemia [80]. In our results, we observed associations with reduced total food intake, as well as lower consumption of simple sugars, carbohydrates, and several micronutrients obtained from the diet, including folic acid, potassium, magnesium, and pantothenic acid. While the reduction in total food intake, particularly of carbohydrates and simple sugars, may be beneficial in the context of obesity prevention, it is important to monitor and address potential micronutrient deficiencies. In particular, chronic potassium deficiency may lead to muscle weakness, cardiac arrhythmias, and, if left untreated, gastrointestinal dysfunction. Therefore, dietary interventions aimed at reducing energy intake should be carefully balanced to ensure adequate micronutrient intake [81]. Magnesium, a cofactor in over 300 enzymatic systems, is critical for energy production by oxidative phosphorylation, glycolysis, and active ion transport [82,83]. Low magnesium levels are associated with chronic conditions such as Alzheimer’s disease, asthma, ADHD, IR, hypertension, migraines, and osteoporosis [84]. Additionally, vitamin B5, or pantothenic acid, is essential for the synthesis of coenzyme A (CoA), which plays a vital role in many catabolic and anabolic reactions [85]. Lower levels of pantothenic acid were detected in some brain regions affected by Alzheimer’s disease compared to controls [86,87]. Thus, personalized nutritional recommendations for carriers of this SNP could include specific foods to ensure adequate micronutrient intake, without raising the glycemic index.

The glucose-dependent insulinotropic polypeptide (GIP) is a gut–brain peptide released from intestinal K cells in response to food intake [88,89]. It stimulates insulin secretion meanwhile inhibits glucagon secretion [89,90,91]. Meanwhile, GWAS showed rs3809770 of *GIP* positively associated with waist circumference, our results link *GIP* rs3809770 to alcohol consumption, as also described by Tsermpini et al. [92], who associated the SNP rs1800437 of this receptor with alcohol consumption, suggesting that the ligand could also play a role in this variable.

The sterol regulatory element-binding protein 1 (SREBP1) is a key nuclear transcription factor involved in lipid synthesis. *SREBF1* SNPs rs2236513/rs2297508/rs4925119 strongly modulated the relationship between cholesterol intake and serum ratio LDL–cholesterol/total cholesterol (*p* < 0.001) [93]. A few years ago, Felder et al. [36] discovered an association between the *SREBF1* SNP rs2297508 and the prevalence of T2DM and adiponectin levels. In our results, the presence of this SNP is associated with a higher dietary intake of iron and riboflavin, which have been shown to protect against diabetic complications by lowering systemic inflammation [94]. It has to be taken into consideration that, in our sample, mainly consisting of obese and overweight individuals, the presence of this SNP may contribute to reduce inflammation in an obesogenic context.

Fatty acid amide hydrolase (FAAH) is one of the most studied enzymes in the metabolism of the endocannabinoid system. It also metabolizes substrates with a role in metabolism and satiety, such as oleoylethanolamide (OEA) and palmitoylethanolamide (PEA). OEA has been shown to reduce food intake and suppression of appetite, effects opposite to those of endocannabinoids. Interestingly, carriers of the *FAAH* rs324420 A allele have been significantly associated with a higher risk of alcohol use disorder (AUD) [95] and substance use disorders, including cannabis dependence. In our results, the *FAAH* rs324420 SNP was positively associated with TNFα levels (*p* = 0.039), in line with other studies where this SNP has been associated with increased BMI and obesity [96,97], as well as markers of systemic inflammation, such as TNFα levels.

Shigemura et al. [98] showed that rs34160967 SNP in *TAS1R1* and rs307377 in *TAS1R3* affected the umami taste sensitivity. Carriers of the *TAS1R1* GG genotype at rs34160967 consumed more fat and total energy compared to the A allele carriers, which has been linked to reduced food palatability [99]. Thus, the rs34160967 polymorphism may have a direct impact on the perception of dietary fats related to the TAS1R1 umami taste receptor subunit [39]. Our data may confirm this trend toward increased total dietary energy, as the presence of this SNP showed an association with the risk of overweight. In addition, in our sample, this SNP was associated with antidepressant consumption, which may be indirectly associated with the tendency of increased risk of overweight.

Fibroblast growth factor 21 (FGF21) is a peptide hormone primarily synthesized and released from the liver. Two genetic studies showed that the common allele in rs838133 SNP was associated with higher intake of carbohydrates and lower intake of proteins and fats, with no effects on total calorie intake [100]; meanwhile, Søberg et al. [43] showed that the preference for carbohydrates intake was specific to sugary products and increase alcohol consumption. Our results could align with this finding, being that rs838133 was associated with an increase in hip circumference, which may be related to sugar-rich diets and fat accumulation in the hip.

**Figure 3:**
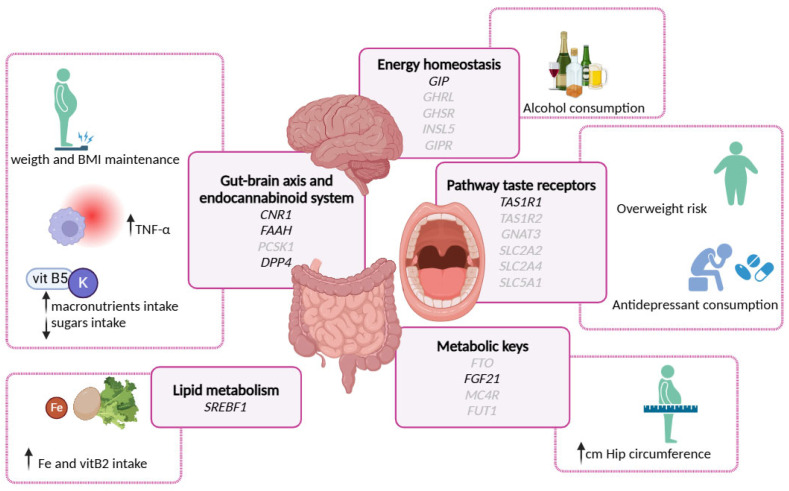
Graphical summary main results of the genotypic associations with various biochemical, anthropometrical, nutrient, and lifestyle variables of the study population.

A summary of the main results of the study are shown in Figure 3.

## 5. Conclusions and Future Directions

Although this is an exploratory analysis, understanding the genetic context of SNPs related to sweet taste sensitivity and the subsequent signaling in the gut–brain axis is crucial for tailoring personalized nutrition to meet individual needs. Our findings underscore the importance of considering genetic variability in sweet taste perception and its related pathways, as these factors can significantly influence metabolic regulation, appetite control, and overall health outcomes. Personalized nutritional recommendations, informed by genetic insights, can help optimize dietary interventions, prevent nutrient deficiencies, and more effectively manage conditions such as obesity and type 2 diabetes. This approach highlights the broader significance of gene–diet–lifestyle interactions in developing targeted and effective health strategies.

The cross-sectional nature of this study limits our ability to establish causality between genetic variants and metabolic outcomes. While significant associations have been identified, they do not determine the direction or causality of these relationships. Therefore, future studies should incorporate longitudinal designs to observe changes over time and establish causal links, providing a more dynamic understanding of the factors influencing overweight and obesity.

This study focuses on individual SNPs; however, exploring gene–gene interactions and polygenic risk factors is essential for understanding complex traits such as obesity. These interactions will offer a more comprehensive view of the genetic architecture underlying these traits.

Additionally, although our sample size of 676 volunteers is substantial, it may still limit the generalizability of our findings to the broader population. The study sample, drawn from volunteers participating in the GENYAL platform at the IMDEA Food Institute, may lack diversity in terms of geographic, ethnic, and socioeconomic backgrounds. Furthermore, the mean BMI of participants exceeds 25, indicating a predominance of overweight and/or obesity. Future studies will incorporate more detailed stratification by BMI categories (e.g., normal weight, overweight, obese) and age groups (e.g., young adults, seniors) to enhance the precision of subgroup analyses and ensure a more representative sample.

By acknowledging these limitations and outlining them as objectives for future research, we aim to strengthen the validity of our findings and contribute valuable insights into the genetic and metabolic factors influencing overweight and obesity.

## Figures and Tables

**Table 1:** Descriptive data of the sample in the study. Variables are shown in stratified subgroups based on BMI values (<25, 25–30 and >30).

**(A) Anthropometry, Vital signs**		**BMI**	**<25**					**BMI**	**25–30**			**BMI**	**>30**			
	**n**	**Min**	**Max**	**Mean**	**SD**	**n**	**Min**	**Max**	**Mean**	**SD**	**n**	**Min**	**Max**	**Mean**	**SD**	
Weight (Kg)	217	42.3	95.2	63.87	8.99	256	144.6	199.5	166.97	9.37	198	145.5	194.5	166.93	9.06	
Height (cm)	215	150	195.8	168.62	8.82	256	52.7	114	77.36	9.57	198	64.3	131.5	92.49	13.01	
BMI	217	18.2	24.9	22.41	1.65	256	25	30	27.57	1.45	198	30.1	43.4	33.03	2.47	
% Total fat	218	7.7	40.8	26.54	8.06	254	11.4	46.7	35.89	8.04	196	26.5	58.7	42.72	7.43	
% Muscle mass	218	23.2	46.7	32.54	5.89	254	21.5	44.4	28.37	4.97	196	18.2	35.9	25.44	4.02	
Visceral fat (BIA)	218	2	9	4.76	1.59	254	4.5	17	8.9	2.37	196	6	24	12.38	4.21	
Waist circumference (cm)	218	62	96.5	77.37	6.71	255	72	114.5	91.41	7.42	198	81.2	132.6	105.03	10.51	
Hip circumference (cm)	150	67	121	97.5	5.74	152	93.8	121	107.99	5.33	153	102	150	117.75	7.83	
SBP (mmHg)	208	49.5	169	116.44	13.82	249	71.5	170	122.08	14.86	194	95	190	128.65	15.22	
DBP (mmHg)	216	27.5	113	71.99	10.91	255	38.5	149	77.18	11.56	197	61	163	82.84	12.84	
**(B) Macronutrient**		**BMI**	**<25**					**BMI**	**25–30**				**BMI**	**>30**		
**intake**																
	**n**	**Min**	**Max**	**Mean**	**SD**	**n**	**Min**	**Max**	**Mean**	**SD**	**n**	**Min**	**Max**	**Mean**	**SD**	**RDA**
Calories (Kcal)	214	1004.6	4406.9	2221.8	609.86	252	986.56	4299.12	2175.7	564.1	195	1016.93	5152.4	2144.6	541.77	1500/2500
Carbohydrates (%TEI)	213	74.04	525.12	214.77	69.9	242	69.12	407.87	208.79	59.33	189	78.04	432.98	201.43	60.92	55
Simple sugars (%TEI)	213	19.78	221	94.3	34.8	245	8.1	229.43	90.6	34.29	190	21.08	195.72	84.44	32.03	10
Proteins (%TEI)	213	45.97	213.71	94.29	28.05	242	39.6	195.81	92.88	24.83	189	28.09	222.63	94.5	24.09	15
Lipids (%TEI)	213	31.1	282.17	99.65	33.19	242	36.55	206.38	96.37	31.73	189	33.55	264.49	96.89	31.14	30
Cholesterol (mg/day)	214	88.31	884.24	341.31	134.71	252	76.4	766.33	332.28	129.69	195	72.41	1013.5	355.29	149.68	<300
SFA (%TEI)	213	10.04	91.22	31.7	11.78	242	10.6	93.43	30.18	11.67	189	8.63	99.59	30.5	11.8	<10
MUFA (%TEI)	213	11.5	113.24	44.19	15.58	242	13.75	101.68	43.17	15.07	189	9.4	99.22	43.24	14.28	15–30
**Micronutrient intake**		**BMI**	**<25**					**BMI**	**25–30**				**BMI**	**>30**		
	**n**	**Min**	**Max**	**Mean**	**SD**	**n**	**Min**	**Max**	**Mean**	**SD**	**n**	**Min**	**Max**	**Mean**	**SD**	**RDA**
Biotin (µg)	198	4.97	75.89	30.51	12.77	221	3.51	145.89	30.17	15.01	182	6.25	59.05	28.1	10.22	50
Potassium (mg)	199	1157.7	7333.7	3205.3	925.6	222	1122.4	6468.2	3136.55	869.22	182	1584.69	5548.17	2997.73	764.71	2000
Magnesium (mg)	199	127.84	726.85	323.03	102.04	222	115.46	672.27	317.27	95.09	182	145.76	598.63	303.28	76.76	375
Iron (mg)	213	7.34	37.85	15.24	4.78	251	4.27	34.31	14.93	4.39	192	7.41	29.24	14.63	4.12	14
Calcium (mg)	213	235	2124	935.5	309.1	251	229	2481.74	902.46	299.83	192	317.03	2151.19	879.67	297.86	800
Folic acid (µg)	213	97.15	1111.8	278.22	112.96	251	85.84	1003.89	288.98	112.8	192	91.53	692.98	276.73	100.74	200
Vit A (µg)	212	122	3600.3	978.69	454.39	250	175.37	19631.4	1079.68	1576.07	192	193.58	37,063.68	1190.02	3173.61	700/900
Vit B1 (mg)	199	0.75	3.77	1.54	0.52	228	0.57	3.16	1.49	0.5	184	0.53	3.09	1.43	0.44	1.1
Vit B2 (mg)	199	0.62	4.63	1.98	0.6	228	0.58	5.03	1.91	0.63	184	0.65	4.84	1.88	0.61	1.4
Vit B3 (mg)	198	14.83	803.49	42.97	55.64	221	16.72	76.6	38.23	10.34	182	12.71	86.77	38.29	10.91	16
Vit B5 (mg)	198	1.46	10.88	5.58	1.46	221	2.13	15.21	5.66	1.72	182	2.44	19.03	5.52	1.79	5
Vit B12 (mg)	199	1.66	50.57	6.8	4.93	228	1.07	74.2	7.3	7.03	184	1.26	42.04	6.81	4.84	2.5
Vit C (mg)	213	25.21	391.77	141.07	68.91	251	12.85	455.76	147.18	75.82	192	18.92	351.58	132.87	71.73	80
Vit D (mg)	213	0.04	24.12	3.87	3.73	251	0.12	43.6	3.56	4.34	192	0.05	29.51	3.38	3.22	5
**(C) Biochemical data**		**BMI**	**<25**					**BMI**	**25–30**				**BMI**	**>30**		
	**n**	**Min**	**Max**	**Mean**	**SD**	**n**	**Min**	**Max**	**Mean**	**SD**	**n**	**Min**	**Max**	**Mean**	**SD**	**RDA**
Glucose (mg/dL)	118	65	121	83.75	9.02	238	62	212.75	86.81	13.2	197	63	170	88.5	12.81	74–115
Cholesterol (mg/dL)	189	124.21	291.2	191.01	32.65	256	118.2	309.5	203.8	36.32	196	123.7	316.7	209.89	38.2	<200
HDL (mg/dL)	189	31.97	94	58.26	12.72	256	31.1	109.6	53.78	12.9	196	21.7	97.3	50.7	11.63	<50
LDL (mg/dL)	189	44.32	196.9	115.39	29.41	254	65.15	214	128.3	31.92	196	62	233	135.08	33.33	<160
Triglycerides (mg/dL)	189	25	200	74.06	32.01	255	26	516	104.89	57.25	196	37	605	115.44	62.76	<150
Insulin (ng/dL)	80	2.4	18.4	5.84	2.66	196	1.8	39.8	7.89	3.8	181	2.7	37.4	11.14	5.45	5–15
Leptin (ng/dL)	31	0.11	88.19	17.21	17.81	15	0.89	55.9	18.91	16.22	197	9	77	19.63	7.22	1–15
GOT (units/L)	162	8	34	18.52	4.89	241	11	45	18.78	5.6	197	6	156	25.02	14.96	<31
GPT (units/L)	162	6	47	16.55	6.29	241	5	89	20.23	11.06	197	0.11	9.76	1.76	1.49	<35
IL6 (ng/L)	28	0.1	6.48	1.97	1.44	99	0.13	66.03	2.22	6.6	97	0.36	14.81	3.11	2.74	0.4–1.4
IL8 (ng/L)	61	0.29	12.81	3.32	2.61	121	0.46	22.89	3.2	3.15	96	0.14	5.7	1.26	0.88	2–10
IL1b (ng/L)	22	0.2	3.21	1.53	0.79	62	0.15	3.67	1.24	0.8	97	0.65	17.43	4.3	2.41	200–500
TNFα (ng/L)	61	0.88	28.84	3.96	3.69	121	0.82	29.43	4.39	3.38	155	87.1	249	153.07	31.19	0.75–5
APOA1 (mg/dL)	36	136	214	164.91	20.6	121	104	317	162.27	31.74	196	63	170	88.5	12.81	120–180
**(D) Eating habits**		**BMI**	**<25**						**BMI**	**25–30**				**BMI**	**>30**	
	**n**	**Min**	**Max**	**Mean**	**SD**	**n**	**Min**	**Max**	**Mean**	**SD**	**n**	**Min**	**Max**	**Mean**	**SD**	
Meals out Mon-Fri	203	0	3	0.68	0.86	234	2	5	3.72	0.77	191	0	5	0.5	0.78	
Meals at home Mon-Fri	203	1	10	3.46	1.23	234	0	4	0.65	0.89	191	0	5	3.45	1.1	
Meals out Sat-Sun	203	0	4	0.77	0.79	234	0	14	3.5	1.47	191	0	4.5	0.6	0.77	
Meals at home Sat-Sun	203	0	6	3.31	1.08	234	0	3	0.59	0.66	191	0	5	3.33	1.11	
Water (mL)	181	0	4500	1442.99	735.08	233	0	3000	1294.15	594.07	196	0	3500	1285.26	704.46	
Glycemic index	198	33.91	98.09	70.05	20.42	227	35.89	119.76	75.68	18.73	184	32.5	118.72	74.84	19.56	
**(E) Other variables related to lifestyle variables**																
Exercise (week)		**n**		**0**		**1**		**2**		**3**		**4**		**>5**		
		647		34%		7.88%		18.39%		16.54%		17.93%		5.26%		
Urination (day)		**n**		**dk/na**		**2**		**3**		**4**		**5**		**>5**		
		639		1.56%		1.25%		8.76%		15.02%		26.29%		47.10%		
Bowel movement		**n**		**dk/na**		**Daily**		**2 days**		**>2 days**						
		639		1.56%		77.15%		14.76%		6.42%						
Alcohol/week		**n**		**0**		**0–5**		**5–10**		**10–15**						
		622		33.12%		49.04%		13.83%		3.05%						
Mental problems		**n**		**Yes**		**No**										
Depression		650		0.77%		99.23%										
Stress		636		25.47%		74.53%										
Anxiety		643		15.71%		84.29%										
Antidepressants		655		1.68%		97.86%										
Smoking		651		13.82%		86.18%										

**Table 2:** Genes and single-nucleotide polymorphisms (SNPs) included in the glucosensing chip.

Gene	SNP	MAF	Functionality	Bibliography
*CNR1*	rs1049353	0.26906 (T)	Synonymous variant	Abdominal adiposity [31], BMI [32]
*DPP4*	rs12617656	0.32893 (C)	Intronic variant	Type 2 Diabetes (DM2) [33]
*GIPR*	rs1800437	0.20971 (C)	Nonsense	Glucose homeostasis [34], obesity [35]
*SREBF1*	rs2297508	0.43149 (C)	Non-coding transcription variant	DM2 prevalence, adiponectin levels [36]
*FAAH*	rs324420	0.199837 (A)	Nonsense	Obesity [37], dyslipemia [38]
*TAS1R1*	rs34160967	0.132269 (A)	Nonsense	Higher energy and fat consumption [39]
	rs731024	0.335981 (A)	Intronic variant	Sugar intake [40]
*GIP*	rs3809770	0.39782 (G)	Upstream variant	Possible DM2 risk [41]
*FGF21*	rs838133	0.44310 (A)	Synonymous variant	Macronutrient and sugar intake [42,43]
	rs838145	0.39468 (G)	Intronic variant	High carbohydrate and calorie intake [42,44]
*SLC5A1*	rs9609429	0.254067 (C)	Upstream variant	Blood pressure [45]
*TAS1R2*	rs12033832	0.32579 (A)	Blood pressure	Blood pressure [26,46]
	rs3935570	0.266728 (T)	Intronic variant	Dental caries [47]
*FTO*	rs11642841	0.397825 (A)	Intronic variant	Obesity [48]
*INSL5*	rs17495511	0.25539 (T)	2KB upstream variant	Blood proteins [49]
*GNAT3*	rs2074673	0.304752 (G)	3′UTR variant	Oral microbiota [40]
*GHRL*	rs27647	0.40810 (C)	Intronic variant	Insulin sensitivity [50]
*FUT1*	rs28400014	0.48936 (G)	2KB upstream variant	Taste measurement [51]
*SLC2A2*	rs5400	0.136075 (A)	Nonsense	DM2 risk [52]
	rs8192675	0.30001 (C)	Intronic variant	Favorable response to DM2 treatment [53]
*SLC2A4*	rs5415	0.292429 (T)	2KB upstream variant	HbA1c levels [54], heart disease risk [55]
	rs5418	0.412875 (G)	5′UTR variant	
*MC4R*	rs571312	0.229418 (A)	No data	BMI and obesity [56]
*GHSR*	rs572169	0.298022 (T)	Synonymous variant	Obesity [57]
*PCSK1*	rs6235	0.26683 (G)	Nonsense	Glucose homeostasis and DM2 [58]

**Table 3:** Genetic distribution and Hardy–Weinberg equilibrium test in the sample. *p* values are shown.

Gene	SNP	Functionality	Ref.A	MAF	Genotype (%)			HWE
					0 (%)	1 (%)	2 (%)	
*CNR1*	rs1049353	Synonymous variant	C	T = 0.26906	56.31	37.25	6.44	0.8719
*DPP4*	rs12617656	Intronic variant	T	C = 0.32893	48.37	40.83	10.8	0.185
*GIPR*	rs1800437	Nonsense	G	C = 0.20971	67.05	29.29	3.66	0.6294
*SREBF1*	rs2297508	Non-coding transcription variant	G	C = 0.43149	36.02	46.98	17	0.5129
*FAAH*	rs324420	Nonsense	C	A = 0.199837	65.1	31.98	2.92	0.2696
*TAS1R1*	rs34160967	Nonsense	G	A = 0.132269	79.37	18.74	1.89	0.1121
	rs731024	Intronic variant	G	A = 0.335981	47.23	40.68	12.09	0.05
*GIP*	rs3809770	Upstream variant	A	G = 0.39782	36.24	45.96	17.8	0.1907
*FGF21*	rs838133	Synonymous variant	G	A = 0.44310	33.5	47.1	19.4	0.2988
	rs838145	Intronic variant	A	G = 0.39468	32.87	50.76	16.37	0.2422
*SLC5A1*	rs9609429	Upstream variant	T	C = 0.254067	59.01	35.91	5.08	0.7787
*TAS1R2*	rs12033832	Synonymous variant	G	A = 0.32579	45.55	44.53	9.92	0.6184
	rs3935570	Intronic variant	G	T = 0.266728	53.16	39.11	7.72	0.7444
*FTO*	rs11642841	Intronic variant	C	A = 0.397825	39.29	47.07	13.65	0.8776
*INSL5*	rs17495511	2KB Upstream variant	C	T = 0.25539	58.64	35.81	5.55	0.9802
*GNAT3*	rs2074673	3′UTR variant	A	G = 0.304752	50.06	41.72	8.22	0.7978
*GHRL*	rs27647	Intronic variant	T	C = 0.40810	34.18	47.72	18.1	0.6071
*FUT1*	rs28400014	2KB Upstream variant	C	G = 0.48936	27.57	51.21	21.22	0.4592
*SLC2A2*	rs5400	Nonsense	G	A = 0.136075	71.05	26.3	2.65	0.8264
	rs8192675	Intronic variant	T	C = 0.30001	46.42	43.4	10.19	0.9666
*SLC2A4*	rs5415	2KB Upstream variant	C	T = 0.292429	53.16	40.51	6.33	0.323
	rs5418	5′UTR variant	A	G = 0.412875	36.35	47.83	15.82	0.9771
*MC4R*	rs571312	No data	C	A = 0.229418	65.41	31.19	3.4	0.771
*GHSR*	rs572169	Synonymous variant	C	T = 0.298022	55.42	37.66	6.93	0.7203
*PCSK1*	rs6235	Nonsense	C	G = 0.26683	60.98	33.88	5.14	0.7226
*PYY*	rs8074783	No data	A	C = 0.4913	40.03	46.42	13.55	0.9807

**Table 4:** Association between the SNPs in the glucosensing chip and anthropometry, biochemistry and lifestyle characteristics.

Gene	SNP	Variable	n	Model	Beta	*p*	*p*_adj_
*CNR1*	rs1049353	Glycemic index	579	Add	−4.23 (−6.8, −1.67)	0.001	0.033
*DPP4*	rs12617656	Total food (g)	564	Add	−132 (−205, −60)	3.56 × 10^−4^	0.009
		Pantothenic acid	576	Add	−0.388 (−0.581, −0.194)	9.39 × 10^−5^	0.002
		Magnesium	578	Add	−19.9 (−30.7, −9.04)	3.47 × 10^−4^	0.009
		Potassium	578	Add	−183 (−283, −83.6)	3.35 × 10^−4^	0.009
		Folic acid	627	Add	−21.8 (−34, −9.72)	4.36 × 10^−4^	0.011
		Carbohydrates	618	Add	−12.3 (−19.1, −5.42)	4.67 × 10^−4^	0.012
		Simple sugars	622	Add	−6.76 (−10.6, −2.96)	5.16 × 10^−4^	0.013
*GIPR*	rs1800437	Legumes	564	Cod	−3.62 (−15.3, 8.05)/60.4 (29.3, 91.5)	4.44 × 10^−4^	0.012
		Physical exercise	621	Add	0.383 (0.144, 0.622)	0.002	0.044
*SREBF1*	rs2297508	Riboflavin	586	Add	0.129 (0.0597, 0.198)	2.72 × 10^−4^	0.007
		Fe	630	Add	0.79 (0.313, 1.27)	0.001	0.031
*FAAH*	rs324420	TNFα	309	Cod	0.312 (−0.446, 1.07)/3.47 (1.59, 5.35)	0.002	0.039
*TAS1R1*	rs34160967	Overweight risk	648	Dom	0.522 (0.345, 0.79)	0.002	0.055
	rs731024	Antidepressants	627	Add	0.102 (0.00569, 0.478)	0.001	0.027
*GIP*	rs3809770	Alcohol	629	Add	2 (0.763, 3.24)	0.002	0.041
*FGF21*	rs838133	Hip circumference	429	Cod	−2.48 (−4.62, −0.344)/2.06 (−0.834, 4.96)	0.002	0.053
*SLC5A1*	rs9609429	Bowel motility	600	Add	0.121 (0.0459, 0.196)	0.002	0.042

## Data Availability

The datasets presented in this article are not readily available because they are part of the GENYAL Platform database for clinical trials in nutrition and health (https://www.food.imdea.org/services/Platform-Clinical-Trials-Nutrition-and-Health, accessed on October–December 2021) database. This database is currently registered as a collection under Spanish rules, which, as a policy of the Centre, will be made public afterwards once the data of the entire expected population are gathered. Requests to access the datasets should be directed to marta.gomezdecedron@alimentacion.imdea.org.

## Appendix A

**List of the studies from where volunteers were included, indicating the acronym of the study, CEI reference and number of individuals from each study:** REDUCOL CEI: HULP-PI935 (n: 44); CAPSA CEI: IMD PI-009 (n: 61); AMC CEI: IMD PI-020 (n: 20); TUTIPASTA CEI: IMD PI-028 (n: 60); SPORT CEI: IMD PI-031 (n: 15); PLATAFORMA CEI: UAM 27-666 (n: 20); INSAOLI CEI: IMD PI:014 (n: 77); NATAC CEI: IMD PI-023 (n: 84): AMC-3 CEI: IMD PI-029 (n: 12); NUTRIPRECISION GULLON CEI: IMD PI-035 (n: 22); SARA CEI: IMD PI-006 (n: 44); FORCANCER CEI: IMD PI-017 (n: 32); GULLON CEI: IMD PI-026 (n: 25); PRIMICIA GENERAL CEI: IMD PI-030 (n: 140); NUTRIPRECISION AMC CEI: IMD PI-036 (n: 20).
